# Understanding of alkaline pretreatment parameters for corn stover enzymatic saccharification

**DOI:** 10.1186/1754-6834-6-8

**Published:** 2013-01-28

**Authors:** Ye Chen, Mark A Stevens, Yongming Zhu, Jason Holmes, Hui Xu

**Affiliations:** 1 Novozymes North America, Franklinton, NC, 27525, USA

**Keywords:** Corn stover, Cellulase, Hemicellulase, Pretreatment, Hydrolysis

## Abstract

**Background:**

Previous research on alkaline pretreatment has mainly focused on optimization of the process parameters to improve substrate digestibility. To achieve satisfactory sugar yield, extremely high chemical loading and enzyme dosages were typically used. Relatively little attention has been paid to reduction of chemical consumption and process waste management, which has proven to be an indispensable component of the bio-refineries. To indicate alkali strength, both alkali concentration in pretreatment solution (g alkali/g pretreatment liquor or g alkali/L pretreatment liquor) and alkali loading based on biomass solids (g alkali/g dry biomass) have been widely used. The dual approaches make it difficult to compare the chemical consumption in different process scenarios while evaluating the cost effectiveness of this pretreatment technology. The current work addresses these issues through pretreatment of corn stover at various combinations of pretreatment conditions. Enzymatic hydrolysis with different enzyme blends was subsequently performed to identify the effects of pretreatment parameters on substrate digestibility as well as process operational and capital costs.

**Results:**

The results showed that sodium hydroxide loading is the most dominant variable for enzymatic digestibility. To reach 70% glucan conversion while avoiding extensive degradation of hemicellulose, approximately 0.08 g NaOH/g corn stover was required. It was also concluded that alkali loading based on total solids (g NaOH/g dry biomass) governs the pretreatment efficiency. Supplementing cellulase with accessory enzymes such as α-arabinofuranosidase and β-xylosidase significantly improved the conversion of the hemicellulose by 6–17%.

**Conclusions:**

The current work presents the impact of alkaline pretreatment parameters on the enzymatic hydrolysis of corn stover as well as the process operational and capital investment costs. The high chemical consumption for alkaline pretreatment technology indicates that the main challenge for commercialization is chemical recovery. However, repurposing or co-locating a biorefinery with a paper mill would be advantageous from an economic point of view.

## Introduction

Alkaline pretreatment is one of several chemical pretreatment technologies that has been intensively investigated. It employs various alkaline reagents including sodium hydroxide [1,2], calcium hydroxide [3,4], potassium hydroxide [5], aqueous ammonia [6,7], ammonia hydroxide [8], and sodium hydroxide in combination with hydrogen peroxide [9,10]. Mechanistically, alkali is believed to cleave hydrolysable linkages in lignin and glycosidic bonds of polysaccharides, which causes a reduction in the degree of polymerization and crystallinity, swelling of the fibers, as well as disruption of the lignin structure [11]. In addition, alkaline saponification of acetyl and uronic ester bonds also improves the enzymatic accessibility of the polysaccharides [12]. The effectiveness of alkaline pretreatment is dependent on the physical structure and chemical composition of the substrate as well as the treatment conditions. In general, alkaline pretreatment is more effective on hardwood, herbaceous crops, and agricultural residues, which have a lower lignin content, than on substrates such as softwood, which contain high amounts of lignin.

Although alkaline pretreatment has been studied on different types of lignocellulosic biomass including switchgrass, corn stover, wheat straw, rice straw, and rice hulls [13], most of the research on alkaline pretreatment has focused on optimization of the process parameters to improve substrate digestibility [13-15]. To achieve this goal, extremely high chemical loading and enzyme dosages were frequently used. Relatively little attention has been paid to process waste management, including chemical recovery and recycle, which has proven to be an indispensable component of the biorefineries [16]. A literature survey also indicates that both alkali concentration in pretreatment solution (g alkali/g pretreatment liquor or g alkali/L pretreatment liquor) and alkali loading based on biomass solids (g alkali/g dry biomass) have been widely used as indicators of alkali strength. The dual approaches make it difficult to compare the chemical consumption in different process scenarios and to evaluate the cost effectiveness of this pretreatment technology. Thus the objectives of this study were to examine the effect of alkaline pretreatment parameters on the digestibility of substrate and to identify whether alkali solution concentration or its dosage on biomass determines hydrolysis yield. The economic feasibility of the alkaline pretreatment process was also evaluated. Corn stover was selected as a model feedstock and a series of alkaline pretreatments were conducted based on a central composite design involving three process variables. Sodium hydroxide was chosen as the pretreatment chemical since it is widely used in the well-established pulp and paper industry. The efficiency of pretreatment was then evaluated by measuring total sugar release from enzymatic hydrolysis of the pretreated substrates.

## Materials and methods

### Feedstock collection and preparation

Corn stover was harvested in the Midwest (United States). Concurrent with the corn (grain) harvest, all residue (leaves stalks and husks) above 12” from the ground was collected. The corn stover residue was then milled to a 6 mm particle size using a Thomas Wiley mill. The moisture content of the corn stover was about 10%. Compositional analysis of the raw corn stover shows that it contains 40.21% glucan, 22.28% xylan, and 19.54% acid insoluble lignin on a dry basis.

### Enzymes

Both Cellic® CTec2 and the experimental accessory enzymes were obtained from Novozymes A/S (Bagsvaerd, Denmark). This particular batch of Cellic® CTec2 had a protein concentration of 141.6 mg protein/g as determined by the bicinchoninic acid (BCA) assay (Pierce, Rockford, Ill.). Cellic® CTec2 and the experimental enzyme cocktails were stored at 4°C and −30°C, respectively, until needed for hydrolysis of pretreated corn stover.

### Alkaline pretreatment

Two sets of alkaline pretreatment studies were conducted to identify: 1) the pretreatment parameters which have the most influence on substrate digestibility and 2) whether the alkali concentration in the aqueous phase or the alkali loading on a dry corn stover basis determines the pretreatment efficiency.

Alkaline pretreatment of corn stover was conducted in a LABOMAT reactor (Type BFA-12, Mathis, Switzerland) with a digitally controlled infrared heating system that has a temperature range of 20-200°C. The instrument is equipped with a variable speed rotary disk and can be programmed to operate with up to 8 stainless steel cylindrical 1 liter beakers simultaneously. Each beaker was loaded with approximately 500 grams of material including corn stover, 50% (w/w) sodium hydroxide solution, and deionized water. Beaker contents were mixed thoroughly to achieve a total solid loading of 11% and the desired alkali loading. Eight stainless steel balls (Dia 10 mm) were added to the beakers to promote more adequate mixing during rotary movement of the beakers. Pretreatment temperature was monitored with a thermocouple inserted through one of the reactor caps. The heat-up time needed to reach target temperature was approximately 10–25 mins, depending on the setpoint pretreatment temperature. Time zero (for pretreatment) was taken to be the time at which the center of the reactor reached the target temperature. After pretreatment, the beakers were immediately quenched in an ice bath for rapid cooling. Corn stover from two replicate beakers treated under similar conditions was recovered, combined, and washed intensively with deionized water to remove soluble phenolics and other degradation products. The washed corn stover was stored at 4°C.

### Enzymatic hydrolysis

Batch enzymatic hydrolysis was performed in 50 mL Nalgene polycarbonate centrifuge tubes (Thermo Scientific, Pittsburgh, PA). Alkali pretreated corn stover was mixed with 50 mM sodium acetate buffer (pH 5.0) supplemented with enzymes as well as 2.5 mg/L lactrol to prevent microbial growth. The final total solids concentration was 10% (w/w). The reaction mixtures (20 g) were agitated in a hybridization incubator (Combi-D24, FINEPCR®, Yang-Chung, Seoul, Korea) at 50°C for 120 hrs. To evaluate pretreatment efficiency as well as the effect of accessory enzymes on hydrolysis performance, pretreated corn stover was hydrolyzed with an enzyme blend at 4 mg protein/g glucan dosage. The enzyme mixture contained 90% protein from Cellic® CTec2, 3.33% protein from arabinofuranosidase which has activity on single substituted arabinose side chain, 3.33% arabinofuranosidase which has activity on double substituted arabinose side chain, and 3.33% β-xylosidase was tested. Pretreated corn stover hydrolyzed with 100% Cellic® CTec2 also at 4 mg protein/g glucan was used as a control. At the end of hydrolysis, 600 μL of hydrolysate were transferred to a Costar Spin-X centrifuge filter tube (Cole-Parmer, Vernon Hills, IL) and filtered through a 0.2 μm nylon filter during centrifugation (14,000 rpm, 20 mins). Supernatant was acidified with 5 μL of 40% (w/v) sulfuric acid to deactivate residual enzyme activity and analyzed by HPLC for sugar concentrations.

### Feedstock compositional analysis and sugar analysis

Total solids content, structural carbohydrate, and lignin content of raw corn stover and alkali pretreated corn stover were analyzed using standard laboratory analytical procedures (LAP) developed by the National Renewable Energy Laboratory (NREL) [17,18]. Sugar samples from compositional analysis were measured using an Agilent 1200 series modular HPLC (Santa Clara, CA) equipped with an Aminex HPX-87P column (Bio-Rad, Richmond, CA), while sugars released from hydrolysis of pretreated corn stover were analyzed using a Rezex ROA-Organic acid H^+^ column (8%) (7.8 × 300 mm) (Phenomenex Inc., Torrance, CA). The methodology was described in detail in Chen et al. [16]. The overall glucan/xylan conversions from hydrolysis were calculated based on sugar concentrations in the enzyme hydrolysis supernatant and composition of the pretreated feedstock using a method similar to that published by Zhu et al. [19].

### Experimental design and statistical analysis

A central composite design was used to reduce the total number of experiments needed to explore the relationship between pretreatment condition and compositional change of pretreated corn stover, as well as its glucan/xylan conversion. The statistical software SAS JMP, version 8 was used for the 3 × 3 central composite design in which 16 pretreatment combinations were derived by altering the three independent variables: alkaline loading, temperature, and time (Table 1) and to analyze the experimental data obtained. The selection of the factorial levels was based on previous studies (data not shown) and the parameters were varied from 60–130°C for temperature, 0.01-0.10 g NaOH/g dry corn stover for chemical dose, and 30–120 mins for pretreatment time. All pretreatment and hydrolysis were performed in duplicate unless otherwise stated. When data have been collected in accordance with the experimental design, the response variable (Y) was fitted to the appropriate empirical equations (second order polynomial regression equations) to identify the key variables:

$$Y=\beta_{o}+\beta_{1}x_{1}+\beta_{2}x_{2}+\beta_{3}x_{3}+\beta_{11}x_{1}^{2}+\beta_{22}x_{2}^{2}+\beta_{33}x_{3}^{2}+\beta_{12}x_{1}x_{2}+\beta_{13}x_{1}x_{3}+\beta_{23}x_{21}x_{3}$$

where the response variable Y represents compositional change of pretreated corn stover or glucan/xylan conversion and the variables x_1_, *x*_2_, and x_3_ correspond to alkaline loading, pretreatment temperature, and time, respectively. The predicted response was therefore correlated to the intercept (β_0_), linear (β_1_, β_2_, β_3_), interaction (β_12_, β_13_, β_23_) and quadratic coefficients (β_11_, β_22_, β_33_) which can be calculated from the experimental data. The quality of fit of the polynomial model equation was expressed by the coefficient of determination. An effect is significant if its p-value is less than 0.05.

**Table 1:** Central composite design of alkaline pretreatment of corn stover

**Sample ID**	**NaOH loading (g NaOH/g dry corn stover)**	**Pretreatment temp (°C)**	**Pretreatment time (min)**
**symbol**	**X_1_**	***X*_2_**	**X_3_**
1	0.052	74	102
2	0.100	95	75
3	0.070	130	75
4	0.070	95	120
5	0.088	116	102
6	0.052	74	48
7 ^a^	0.070	95	75
8	0.088	116	48
9	0.040	95	75
10	0.070	60	75
11	0.088	74	102
12	0.052	116	102
13	0.070	95	30
14	0.052	116	48
15	0.088	74	48
16 ^a^	0.070	95	75

^a^ Center point of the central composite design.

## Results and discussion

### Alkaline pretreatment of corn stover

Table 2 summarizes the compositional change of corn stover following pretreatment. During alkaline pretreatment, the cleavage of hydrolyzable linkages such as α- and β- aryl ethers in lignin and glycosidic bonds in carbohydrates constitute the primary reactions that lead to the dissolution of lignin and carbohydrate with lower alkali stability [20]. More than 95% of the cellulose in corn stover was preserved in alkaline pretreatment, which can be explained by the low reactivity of cellulose with alkali and also its high crystallinity [20,21]. Dissolution of hemicellulose and lignin, however, varied significantly depending on the pretreatment conditions (Table 2). Table 3 shows the effect of the pretreatment parameters on xylan recovery as well as on delignification. The statistical analysis indicates that among the variables that have a statistically significant effect on lignin removal from corn stover (three first-order effects, three second-order effects, and one interaction effect), NaOH loading had the most significant impact (regression coefficient β_1_ = 8.73), indicating the highest sensitivity of lignin content to alkali charge. When alkali loading increased from 0.04 to 0.1 g/g corn stover, the residual lignin decreased from 67.5 to 20.1% (Table 2). Although pretreatment at high alkali loading, temperature, and longer residence time can maximize delignification and therefore improve substrate digestibility, high severity pretreatment conditions may also lead to undesired sugar loss through dissolution and degradation of hemicellulose. Similar to lignin degradation, depolymerization of hemicellulose is also significantly affected by the three parameters with alkali loading having the greatest effect (regression coefficient β_1_ = −2.922). Xylan degradation increased by 20% when NaOH loading increased from 0.04 to 0.1 g/g corn stover (Table 2). These reaction mechanisms imply that a balance between extent of delignification and preservation of carbohydrate has to be established in order to achieve maximum overall sugar yield.

**Table 2:** Composition of washed pretreated corn stover solids

**Temp (°C)**	**NaOH loading (g NaOH/g corn stover)**	**Time (mins)**	**Composition (%) ^a,b^**			**Recovery (%) ^b,c^**			
			**Glucan**	**Xylan**	**AIL**	**Glucan**	**Xylan**	**AIL**	**Total**
74	0.052	102	48.11	25.75	14.44	96.16	92.88	59.41	80.38
95	0.100	75	61.20	28.20	6.42	93.25	77.53	20.12	61.26
130	0.070	75	57.85	25.98	9.74	95.75	77.61	33.17	66.55
95	0.070	120	55.46	27.20	8.98	96.47	85.39	32.14	69.95
116	0.088	102	59.53	26.91	5.36	95.20	77.67	17.65	64.30
74	0.052	48	46.63	25.31	14.84	96.66	94.69	63.28	83.35
95 ^d^	0.070	75	54.60	26.16	8.37	97.64	84.44	30.81	71.91
116	0.088	48	60.24	27.53	4.74	96.89	79.92	15.69	64.68
95	0.040	75	45.25	25.06	15.20	97.69	97.64	67.50	86.80
60	0.070	75	48.48	25.18	12.19	97.55	91.44	50.47	80.90
74	0.088	102	54.03	25.64	8.06	95.83	82.07	29.40	71.31
116	0.052	102	50.67	24.68	13.36	95.70	84.13	51.94	75.95
95	0.070	30	51.60	26.00	11.48	96.43	87.68	44.13	75.14
116	0.052	48	49.43	25.03	14.07	96.46	88.13	56.50	78.46
74	0.088	48	54.34	25.54	9.35	97.13	82.39	34.38	71.87
95 ^d^	0.070	75	54.22	26.33	8.21	97.81	84.29	30.26	71.59

^a^ The composition of insoluble solids are based on oven-dry weight. ^b^ Values are expressed as averages of three replicate samples. Coefficient of variation (CV) is below 2.1 %. ^c^ Values were calculated based on the total recovery, composition of alkaline pretreated, as well as that of raw corn stover. ^d^ Center point of the central composite design.

**Table 3:** **Statistical analysis of the effects of pretreatment parameters on corn stover xylan recovery and delignification**^**a**^

	**Xylan recovery**		**Lignin removal**	
R^2^	0.942		0.985	
Prob. > F	< 0.0001*		< 0.0001*	
**Terms**	**Estimate**	***p *value**	**Estimate**	***p *value**
NaOH loading	−2.922	< 0.0001*	8.730	< 0.0001*
Temperature	−0.002	< 0.0001*	0.003	< 0.0001*
Time	−0.0003	0.015*	0.001	< 0.0001*
NaOH loading × temperature	0.028	0.027*	0.053	0.003*
NaOH loading × time	0.008	0.374	−0.014	0.272
Temperature × time	−9.09 × e^-6^	0.258	−1.376 × e^-5^	0.208
NaOH loading × NaOH loading	31.764	0.0218*	−137.865	< 0.0001*
Temperature × temperature	−1.62 × e^-6^	0.865	−8.50 × e^-5^	< 0.0001*
Time × time	8.93 × e^-6^	0.133	−3.325 × e^-5^	< 0.0003*

^a^ Numbers with asterisk indicate that the term has a significant effect at 95 % confidence interval.

### Enzymatic hydrolysis of pretreated corn stover

Glucan and xylan conversions for hydrolysis and for the overall process (pretreatment and hydrolysis) are presented in Table 4. Statistical analysis of the hydrolysis data, which examines the relationship between pretreatment parameters and conversions, is summarized in Table 5. All four models have R^2^ values between 0.91 and 0.97, indicating that a large fraction of the variation in responses can be accounted for by the independent variables. The analysis of variance also showed that the second order polynomial regression models are highly significant (p value < 0.0001) (Table 5).

**Table 4:** **Enzymatic hydrolysis of alkaline pretreated corn stover**^**a**^

**Temp (°C)**	**NaOH loading (g NaOH/g corn stover)**	**Time (mins)**	**Hydrolysis yield^b^**		**Pretreatment and hydrolysis yield^c^**		**Enzyme accessibility^d^**	
			**Glucan**	**Xylan**	**Glucan**	**Xylan**	**Glucan**	**Xylan**
74	0.052	102	47.35%	45.56%	45.53%	42.32%	56.79%	55.30%
95	0.100	75	82.31%	75.67%	76.75%	58.67%	88.19%	79.84%
130	0.070	75	59.83%	67.21%	57.28%	52.16%	88.42%	79.87%
95	0.070	120	69.63%	70.47%	67.17%	60.17%	83.98%	77.39%
116	0.088	102	82.63%	76.62%	78.66%	59.51%	94.12%	84.55%
74	0.052	48	49.29%	42.53%	47.65%	40.27%	64.21%	58.07%
95 ^d^	0.070	75	68.42%	72.94%	66.81%	61.59%	89.75%	80.58%
116	0.088	48	82.32%	79.22%	79.76%	63.32%	91.42%	82.03%
95	0.040	75	47.18%	38.04%	46.09%	37.14%	59.40%	50.27%
60	0.070	75	55.72%	53.82%	54.35%	49.22%	66.25%	62.45%
74	0.088	102	75.71%	73.24%	72.55%	60.11%	90.89%	80.43%
116	0.052	102	61.11%	61.76%	58.49%	51.96%	95.57%	83.29%
95	0.070	30	68.01%	64.40%	65.59%	56.46%	86.79%	76.17%
116	0.052	48	55.42%	60.37%	53.45%	53.20%	80.88%	71.78%
74	0.088	48	70.54%	72.13%	68.51%	59.43%	88.90%	80.73%
95 ^d^	0.070	75	68.71%	73.00%	66.99%	61.95%	89.95%	81.20%

^a^ Values are expressed as averages of two replicate samples. Coefficient of variation (CV) is below 2.5%.^b^ Glucose and xylose yield was calculated based on glucan and xylan content in pretreated and washed corn stover. ^c^ glucose and xylose yield was calculated based on glucan and xylan content in raw corn stover. ^d^ Glucan (or xylan) enzyme accessibility was defined as the fraction of glucan (or xylan) in biomass that can be converted to monomeric sugars by enzyme activities. They were obtained by measuring glucan or xylan conversions at an extremely high enzyme dosage (50 mg Cellic® CTec2/g glucan).

**Table 5:** **Statistical analysis of the effects of pretreatment parameters on corn stover hydrolysis**^a^

	**Glucose (hydrolysis)**		**Xylose (hydrolysis)**		**Glucose (overall)**		**Xylose (overall)**	
R^2^	0.951		0.966		0.914		0.939	
Prob > F	< 0.0001*		< 0.0001*		< 0.0001*		< 0.0001*	
**Terms**	**Estimate**	***p*value**	**estimate**	***p*value**	**Estimate**	***p*value**	**Estimate**	***p*value**
NaOH loading	6.417	< 0.0001*	5.084	< 0.0001*	5.962	< 0.0001*	2.987	< 0.0001*
Temperature	0.002	< 0.0001*	0.002	< 0.0001*	0.001	< 0.0001*	0.001	< 0.0001*
Time	0.0003	0.159	0.0003	0.0592	0.0002	0.336	9.604 × e^-5^	0.467
NaOH loading × temperature	−0.004	0.852	−0.052	0.0017*	−0.004	0.835	−0.042	0.0022*
NaOH loading × time	0.004	0.786	−0.006	0.594	0.0001	0.998	−0.003	0.743
Temperature × time	6.139 × e^-6^	0.663	6.798 × e^-6^	0.491	4.46 × e^-6^	0.763	3.804 × e^-7^	0.948
NaOH loading × NaOH loading	−26.181	0.263	−86.943	< 0.0001*	−43.260	0.086	−76.638	< 0.0001*
Temperature × temperature	−7.616 × e^-5^	0.0002*	−6.44 × e^-5^	< 0.0001*	−7.753 × e^-5^	0.0002*	−5.918 × e^-6^	< 0.0001*
Time × time	8.50 × e^-6^	0.411	−4.701 × e^-6^	0.514	5.53 × 10^-6^	0.628	3.898 × e^-7^	0.948

^a^ Numbers with asterisk indicate that the term has a significant effect at 95 % confidence interval.

Alkali loading and temperature have a significant effect on glucan conversion, which is consistent with previous studies investigating alkaline pretreatment of various lignocellulosic feedstocks [13,22]. Glucan conversion during hydrolysis is positively correlated with NaOH loading. An increase of NaOH loading from 0.04 to 0.1 g/g corn stover improved glucan conversion by 35% during hydrolysis (Table 4). Since more than 95% of the original glucan was preserved in the solid fraction following pretreatment, this increase was also reflected in the overall process yield. To reach 70% overall glucan conversion at 4 mg protein/g glucan enzyme dose, approximately 0.08 g NaOH/g corn stover was required.

All the linear and quadratic model terms that include alkali loading and temperature have a significant effect on xylan conversion during hydrolysis and for the overall process (Table 5). Among linear terms, NaOH loading had the greatest effect on the responses while this variable had a significant interaction with temperature (p-value < 0.05). Xylan conversion during hydrolysis is positively correlated with NaOH loading. An increase in NaOH loading from 0.04 to 0.1 g/g corn stover improved xylan conversion by 37% during hydrolysis (Table 4). However, alkali delignification processes are usually accompanied by dissolution and degradation of hemicellulose [21]. When NaOH loading exceeded a certain limit (approximately 0.08 g/g corn stover), the substantial loss of carbohydrates during pretreatment can offset increased substrate digestibility.

Temperature is the second most important parameter affecting hydrolysis conversion. The models indicate that the optimal temperature ranges are 103–106°C and 93–97°C for glucose and xylose release, respectively. An increase in temperature accelerates delignification. However, severe pretreatment conditions can lead to lignin condensation reactions that form carbon-carbon bonds between lignin subunits, thereby limiting its removal and consequently reducing glucan/xylan conversion [23]. In addition, higher temperatures also increase carbohydrate loss through random chain cleavage as well as peeling reactions, which can greatly reduce the sugar yield from the overall process [24].

Although alkaline pretreatment and chemical pulping share many similarities in reaction chemistry and substrate physicochemical changes, the desired outcomes from pretreatment and pulping are very different. The purpose of chemical pulping is to remove lignin and improve paper strength. Most of the pulp mills, with the exception of those practicing high yield pulping, delignify biomass extensively to save on bleaching chemical costs. The final kappa number for unbleached pulp is typically between 15–30, which corresponds to 2.5–4.5% lignin content or more than 90% delignification [25]. As a result, there is a significant loss of hemicellulose due to peeling reactions and the overall pulp yield is in the range of 45–50% [26]. In the case of biomass pretreatment, the purpose is to depolymerize cellulose/hemicellulose into fermentable sugars; high carbohydrate yields are essential for economic viability. The conditions used in biomass pretreatment are much milder than pulping including lower alkali charge, lower temperatures and shorter residence times. Consequently, the pulp yield after pretreatment is significantly higher (61–72%) than that of the chemical pulping process.

### Impact of hemicellulase supplement

**Figure 1:**
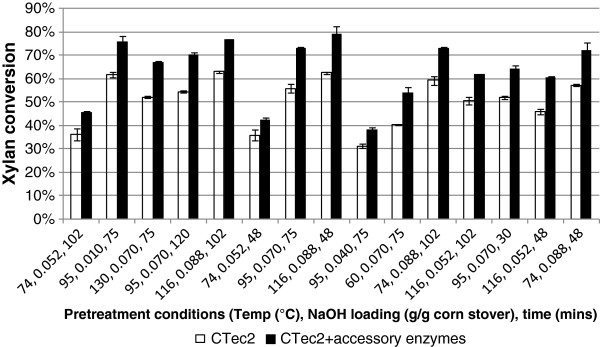
Improvement of xylan conversion of alkaline pretreated corn stover by accessory hemicellulases.

Alkaline pretreated corn stover had a xylan content of 25–28%, which implies that hemicellulases should be indispensable components in the hydrolysis of biomass pretreated under alkaline conditions. To efficiently hydrolyze the xylan and xylooligomers remaining after pretreatment, CTec2 was supplemented with a 3% (based on protein dose) replacement of an experimental hemicellulase mixture which contained accessory enzymes such as arabinofuranosidases and β-xylosidase. Hydrolysis was conducted with 4 mg protein/g glucan enzyme dose at 10% total solids loading for 120 hrs. Alkaline pretreated corn stover hydrolyzed with CTec2 only was used as the control. Supplementation of CTec2 with accessory hemicellulases only marginally increased the glucan to glucose conversion by 0–2% (data not shown). The relatively small improvement observed in this study can be attributed to the presence of hemicellulase activities in CTec2. These hemicellulases help remove hemicellulose that physically blocks access to cellulose by cellulase [27]. Hemicellulases also contribute to the decrease in the concentrations of high molecular weight xylooligomers, which have been reported to be highly inhibitory towards cellulase activities [28]. On the other hand, the conversion of xylan to xylose was significantly enhanced by supplementation with accessory enzymes. Depending on the pretreatment conditions, corn stover samples hydrolyzed with CTec2 supplemented with accessory enzymes had xylan conversions that were 6–17% higher than their respective controls (Figure 1). The effect was less pronounced for substrate pretreated with lower NaOH loadings (0.040 and 0.052 g NaOH/g corn stover), possibly due to the fact that their poor accessibility limits enzyme-substrate interaction (Table 4). The higher xylan to xylose yield obtained for hydrolysis with Cellic® CTec2 supplemented with accessory enzymes can be explained as follows. Complete hydrolysis of xylan requires synergistic effect of endo-β-1,4 xylanase, β-xylosidase on xylan backbone and accessory enzymes for hydrolyzing various substituted xylans [29]. However, many xylanases are not capable of cleaving glycosidic bonds between xylose units that are substituted [30]. α-arabinofuranosidase and β-arabinofuranosidase remove the arabinose substituents from the xylan backbone, as indicated by the 1- to 4-fold increase in arabinose concentration in the hydrolysate compared to the controls (data not shown). Removal of side chains allows better access by the xylanase to the linkage between backbone components of the polysaccharide; In addition, β-xylosidase acts synergistically with xylanases and releases xylose monomers from xylobiose and short chain xylooligosaccharides, which contributes to the higher xylan to xylose conversion [28].

### Effect of alkali solution concentration and biomass alkali loading

**Figure 2:**
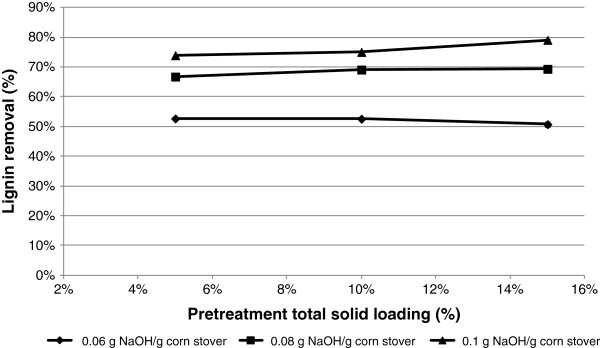
Effect of alkali solution concentration and biomass alkali loading on delignification of corn stover.

In previous studies on alkaline pretreatment, both alkali solution concentration (g alkali/g pretreatment liquor) and biomass alkali loading (g alkali/g biomass) are used as indicators of chemical strength [14,15]. To distinguish the effects of solution strength vs. biomass alkali loading on biomass digestibility, pretreatment of corn stover was conducted at 0.05 g, 0.10 g, and 0.15 g dry corn stover/g slurry. At each solid loading, three NaOH dosages (0.06, 0.08, and 0.10 g NaOH/g corn stover) were used to compare the pretreatment efficiency. In the experiment, pretreatment temperature and residence time were maintained at 90°C and 120 mins, respectively. Compositional analysis of the corn stover pretreated under the 9 different test conditions was performed; delignification of the pretreated corn stover is shown in Figure 2. The extent of delignification was closely correlated with biomass alkali loading. For a given NaOH loading based on corn stover dry weight, lignin removal was relatively stable regardless of the total solids content during pretreatment.

**Figure 3:**
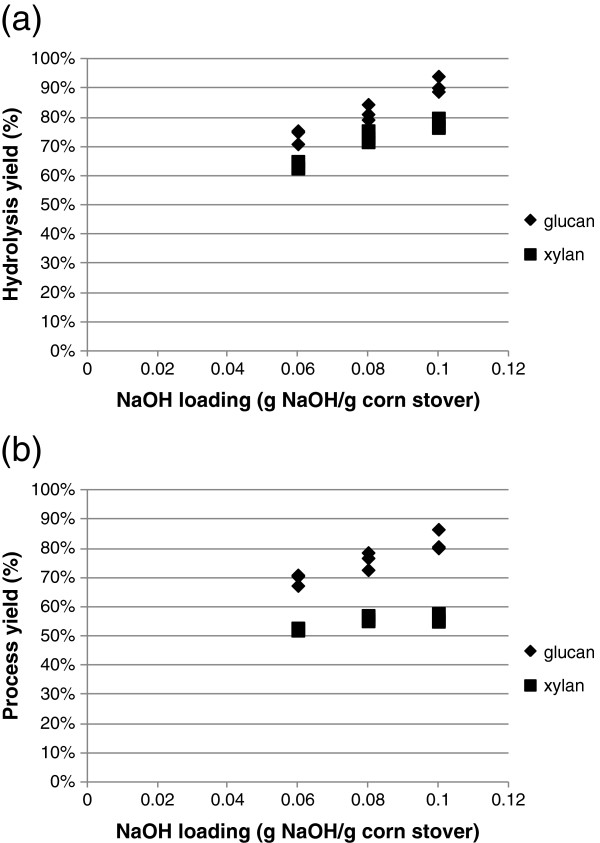
**Relationship between biomass alkali loading and (a) glucan and xylan conversion during hydrolysis and (b) glucan and xylan conversion for the combined processes of pretreatment and hydrolysis.** Hydrolysis of pretreated corn stover was conducted at 8.5% total solids level.

**Figure 4:**
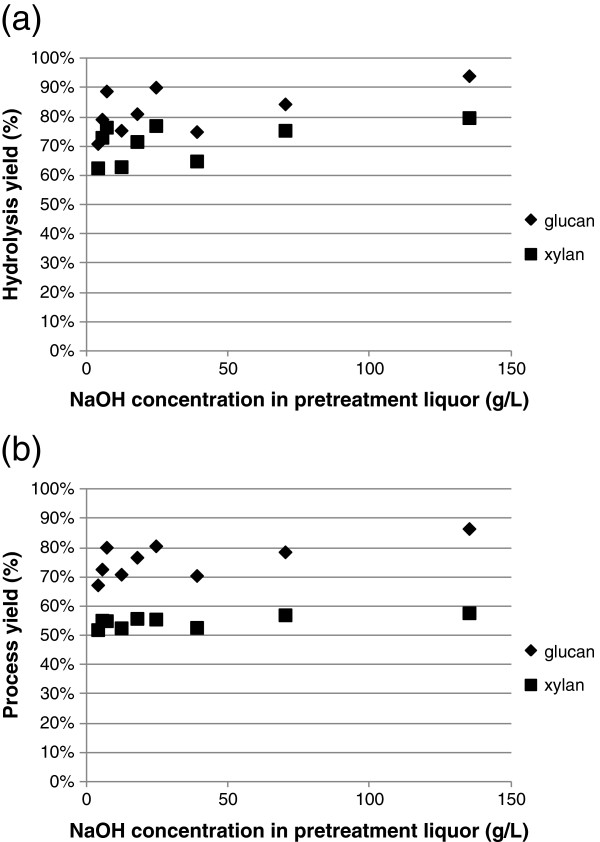
**Relationship between alkali solution concentration and (a) glucan and xylan conversion in hydrolysis and (b) glucan and xylan conversion for the combined processes of pretreatment and hydrolysis.** Hydrolysis of pretreated corn stover was conducted at 8.5 % total solids level.

The pretreated corn stover was washed with deionized water and hydrolyzed with Cellic® CTec2 at 4 mg protein/g glucan for 120 hrs at 8.5% total solids loading. Figure 3 shows the effect of biomass alkali loading on glucose and xylose yields for hydrolysis (Figure 3a) and also for the combined pretreatment and hydrolysis processes (Figure 3b). The effect of alkali solution concentration is shown in Figure 4. Essentially, the enzymatic digestibility of the corn stover correlates better with biomass alkali loading than with alkali solution concentration. Glucose/xylose concentrations in the hydrolysate increased as the alkali charge on dry corn stover increased (Figure 3). On the other hand, no correlation can be established between glucan/xylan conversion and NaOH solution concentration (Figure 4).

Among the three fractions that constitute lignocellulosic materials, cellulose is relatively stable under alkaline conditions due to its high degree of polymerization and high crystallinity. However, hemicellulose is more labile and a significant loss of hemicellulose can occur as a consequence of the dissolution and peeling of undissolved polysaccharides. In addition, alkaline saponification of acetyl and uronic ester groups in hemicellulose proceeds readily and contributes significantly to their partial crystallization [31]. The major reactions that lead to the removal of lignin are the cleavage of α- and β- ether bonds in phenolic units and of β- ether linkages in non-phenolic units. In these reactions, NaOH participates in the ionization of C1 and/or C2 hydroxyl groups on monosaccharide rings, free phenolic hydroxyl groups, and hydroxyl groups at α- or γ- position in lignin monomers. Because NaOH is consumed as these reactions proceed [20], it stands to reason that NaOH loading on dry corn stover is more crucial in determining substrate digestibility than is alkali solution concentration. Based on this study, it can also be concluded that a reduction in chemical consumption is unlikely to be realized simply by increasing the solids loading in the pretreatment reactor.

### Potential of alkaline pretreatment technology

Alkaline pretreatment has a unique application in many integrated biorefineries where value added products, other than ethanol, are produced from lignocellulosics. The advantage of this pretreatment technology lies in the fact that it would create a washed clean substrate which is highly digestible and rich in cellulose and xylan. After enzymatic hydrolysis, a relatively clean sugar stream (both xylose and glucose) could be obtained at reasonably high yield and economically relevant enzyme dose. For processes that are highly sensitive to impurities (inhibitors, salts), alkaline pretreatment is certainly a better choice.

Since alkali loading is the most crucial parameter affecting hydrolysis efficiency and alkali loading on dry biomass governs the digestibility of pretreated corn stover, chemical cost becomes one of the major components of the operating cost as well as total capital investment. For a biomass-to-ethanol plant that has a capacity of 50 million gallons of ethanol per year (processing 2,205 dry ton corn stover per day) [32], approximately 176 tons of NaOH is required per day for pretreatment given the fact that 0.08 g NaOH/g corn stover is needed to reach satisfactory glucan and xylan conversions. The black liquor generated during alkaline pretreatment has to be treated before it can be recycled or released to the environment. Spent chemicals from alkaline processes can be separated from biomass by washing and regenerated through well-established lime kiln technology. The black liquor is concentrated in evaporators to form concentrated black liquor (65–80% solids) which can then be combusted in a recovery boiler to generate sodium carbonate from inorganic sodium. The sodium carbonate salt is subsequently dissolved in water and sent to a causticizing plant to regenerate NaOH by contact with slaked lime. The resulting calcium carbonate is filtered off and returned to a lime kiln where burnt lime is produced, slaked and returned to the causticizer [33,34]. The estimated capital cost of such a chemical recovery system is approximately $121.7–242.1 million [35,36]. Depending on the system installed, this cost may exceed the total equipment cost ($232 million) proposed by the National Renewable Energy Laboratory for a lignocellulosic ethanol plant using dilute acid pretreatment [32]. Therefore, from an economic point of view, an alkali-based biorefinery is less economically attractive unless the cost of chemical recovery can be significantly reduced or, alternatively, low cost recovery systems can be identified and commercialized. A great opportunity to implement alkaline pretreatment process while significantly reducing capital investment would be the repurposing of existing Kraft paper mills to bioethanol plants [37]. Repurposing can take advantage of proven manufacturing infrastructure, existing skilled operating personnel, and an established biomass supply chain [38]. Another possibility would be to co-locate bio-ethanol plants with existing pulp mills that have excess capacity in their chemical recovery systems, such that black liquor produced from pretreatment could be regenerated by nearby pulp mills. However, a thorough energy and economic assessment of a given integrated biorefinery processes is still needed to determine its economical feasibility and to establish the most appropriate operating conditions.

## Conclusions

The effect of pretreatment parameters on enzymatic hydrolysis of corn stover was investigated. It was concluded that the NaOH loading is the most dominant variable for enzymatic digestibility. Although alkali concentration (g NaOH/g pretreatment liquid) has been widely used as an indication of alkali strength in the literature, the experimental results suggest that alkali loading based on total solids (g NaOH/g dry biomass) governs the pretreatment efficiency. Supplementing cellulase with accessory enzymes such as α-arabinofuranosidases and β-xylosidase significantly improved the conversion of the hemicellulose by 6–17%. High chemical consumption can be one of the major hurdles for the commercialization of a biorefinery using alkaline pretreatment technology. However, repurposing or co-locating biorefinery with a paper mill can be a strategy to lower the operating cost as well as total capital investment.

## Abbreviations

BCA: Bicinchoninic acid; LAP: Laboratory analytical procedures; NREL: National renewable energy laboratory.

## Competing interests

The authors declare that they have no competing interests.

## Authors’ contributions

YC participated in the design of experiments, conducted the work presented here, performed the statistical analysis and drafted the manuscript. YZ participated in the experiments design, performed the work, and helped draft the manuscript. MAS and JH conducted the pretreatment and hydrolysis experiments. HX conceived of the study, supervised the work, and assisted in drafting the manuscript. All authors read and approved of the final manuscript.

## References

[B1] Carrillo F Lis M J Colom X López-Mesasa M Valldeperas J Effect of alkali pretreatment on cellulase hydrolyiss of wheat straw: Kinetic study Process Biochem 2005 40 3360 4 10.1016/j.procbio.2005.03.003

[B2] Silverstein RA Chen Y Sharma-Shivappa RR Boyette MD Osborne J A comparison of chemical pretreatment methods for improving saccharification of cotton stalks Bioresource Technol 2007 98 3000 11 10.1016/j.biortech.2006.10.022 17158046

[B3] Chang VS Nagwani M Kim CH Holtzapple MT Oxidative lime pretreatment of high-lignin biomass - poplar wood and newspaper Appl Biochem Biotechnol 2001 94 1 28 10.1385/ABAB:94:1:01 11393353

[B4] Kaar WE Holtzapple MT Using lime pretreatment to facilitate the enzymatic hydrolysis of corn stover Biomass Bioenergy 2000 18 189 99 10.1016/S0961-9534(99)00091-4

[B5] Chang VS Holtzapple MT Fundamental factors affecting biomass enzymatic reactivity Appl Biochem Biotech 2000 84-86 5 37 10.1385/abab:84-86:1-9:5 10849776

[B6] Foster BL Dale BE Doran-Peterson JB Enzymatic hydrolysis of ammonia-treated sugar beet pulp Appl Biochem Biotechnol 2001 91/93 269 82 10.1385/ABAB:91-93:1-9:269 11963856

[B7] Kim TH Kim JS Sunwoo C Lee YY Petreatment of corn stover by aqueous ammonia Bioresource Technol 2003 90 39 47 10.1016/S0960-8524(03)00097-X 12835055

[B8] Prior BA Day DF Hydrolysis of ammonia-pretreated sugar cane bagasse with cellulase, beta-glucosidase, and hemicellulase preparations Appl Biochem Biotechnol 2008 146 151 64 10.1007/s12010-007-8084-0 18421595

[B9] Saha BC Cotta MA Ethanol production from alkaline peroxide pretreated enzymatically saccharified wheat straw Biotechnol Prog 2006 22 449 53 10.1021/bp050310r 16599561

[B10] Saha BC Cotta MA Enzymatic saccharification and fermentation of alkaline peroxide retreated rice hulls to ethanol Enzyme Microb Technol 2007 41 528 32 10.1016/j.enzmictec.2007.04.006

[B11] Hsu TA In Handbook on bioethanol, production and utilization Pretreatment of biomass 1996 Washington DC: Taylor and Francis: Edited by Wyman, CE 179 212

[B12] Zhang YHP Lynd LR Toward an aggregated understanding of enzymatic hydrolysis of cellulose: noncomplexed cellulase systems Biotechnol Bioeng 2004 88 797 824 10.1002/bit.20282 15538721

[B13] Cheng Y-S Zheng Y Yu C-W Dooley TM Jenkins BM VanderGheynst JS Evaluation of high solids alkaline pretreatment of rice straw Appl Biochem Biotechnol 2010 162 1768 84 10.1007/s12010-010-8958-4 20440580 PMC2929346

[B14] Chen BY Chen SW Wang HT Use of different alkaline pretreatments and enzyme models to improve low-cost cellulosic biomass conversion Biomass Bioenergy 2012 39 182 91

[B15] McIntosh S Vancov T Optimization of dilute alkaline pretreatment for enzymatic Saccharification of wheat straw Biomass Bioenergy 2011 35 3094 103 10.1016/j.biombioe.2011.04.018

[B16] Chen Y Stevens MA Zhu YM Holmes J Moxley G Xu H Reducing acid in dilute acid pretreatment and the impact on enzymatic Saccharification J Ind Microbiol Biotechnol 2012 39 5 691 700 10.1007/s10295-011-1068-7 22167347

[B17] Sluiter A Hyman D Payne C Wolfe J Determination of total solids in biomass and total dissolved solids in liquid Process Samples[http://www.nrel.gov/biomass/pdfs/42621.pdf]

[B18] Sluiter A Hames B Ruiz R Scarlata C Sluiter J Templeton D Determination of structural carbohydrates and lignin in biomass[http://www.nrel.gov/biomass/pdfs/42618.pdf]

[B19] Zhu Y Malten M Torry-Smith M McMillan JD Stickel JJ Calculating sugar yields in high solids hydrolysis of biomass Bioresource Technol 2011 102 3 2897 903 10.1016/j.biortech.2010.10.134 21109427

[B20] Lai YZ In Wood and Cellulose Chemistry 2nd edition Chemical Degradation 1991 New York: Marcel Dekker Inc: Edited by Hon DNS and Shiraishi N 455 73

[B21] Gupta R Lee YY Investigation of biomass degradation mechanism in pretreatment of switchgrass by aqueous ammonia and sodium hydroxide Bioresource Technol 2010 101 8185 91 10.1016/j.biortech.2010.05.039 20639115

[B22] Saha BC Cotta MA Lime pretreatment, enzymatic saccharification, and fermentation of rice hulls to ethanol Biomass Bioenergy 2008 32 10 971 7 10.1016/j.biombioe.2008.01.014 15932261

[B23] Pan XJ Zhang X Gregg DJ Saddler JN Enhanced enzymatic hydrolysis of steam-exploded Douglas fir wood by alkali-oxygen post-treatment Appl Biochem Biotechnol 2004 113-116 1103 14 15054256 10.1385/abab:115:1-3:1103

[B24] McDonough TJ In Pulp Bleaching—Principles and Practice Oxygen delignification 1996 DW, Atlanta: TAPPI: Edited by Dence CW and Reeve 213 39

[B25] Chakar FS Ragauskas AJ Review of current and future softwood kraft lignin process chemistry Ind Crop Prod 2004 20 131 41 10.1016/j.indcrop.2004.04.016

[B26] Sjöholma E Gustafssona K Bertholda F Colmsjöb A Influence of the carbohydrate composition on the molecular weight distribution of kraft pulps Carbohyd Polym 2000 41 1 1 7 10.1016/S0144-8617(99)00066-1

[B27] Jeoh T Ishizawa CI Davis MF Himmel ME Adney WS Johnson DK Cellulase digestibility of pretreated biomass is limited by cellulose accessibility Biotechnol Bioeng 2007 98 112 22 10.1002/bit.21408 17335064

[B28] Qing Q Yang B Wyman CE Xylooligomers are strong inhibitors of cellulose hydrolysis by enzymes Bioresource Technol 2010 101 9624 30 10.1016/j.biortech.2010.06.137 20708404

[B29] Buchmann SL McCarthy AJ Purification and cooperative acidity of enzymes constituting the xylan-degrading system of Thermomosospora fusca Appl Environ Microbiol 1991 57 2121 30 16348531 10.1128/aem.57.8.2121-2130.1991 PMC183538

[B30] Lee SF Forsberg CW Purification and characterization of an α-arabinofuranosidase from Clostridium acetobutylicum ATCC 824 Can J Microbiol 1987 33 1011 16 10.1139/m87-178 PMC203729 16347312

[B31] Rydholm SA Pulping processes 1965 New York: Wiley-Interscience

[B32] Humbird D Davis R Tao L Kinchin C Hsu D Aden A Schoen P Lukas J Olthof B Worley M Sexton D Dudgeon D Process design and economics for biochemical conversion of lignocellulosic biomass to ethanol Dilute-acid pretreatment and enzymatic hydrolysis of corn stover [http://www.nrel.gov/biomass/pdfs/47764.pdf]

[B33] Cantrell J Simulation of kraft black liquor gasification - A comparative look at performance and economics TAPPI J 2001 84 6 71 71

[B34] Hamaguchi M Vakkilainen EK Influence of chlorine and potassium on operation and design of chemical recovery equipment TAPPI J 2011 10 1 33 9

[B35] Fallavollita JA Avedesiam MM Mujumdar AS Kraft black liquor recovery in a fluidized bed: part I - a review Can J Chem Eng 1987 65 5 812 17 10.1002/cjce.5450650515

[B36] Katofsky R Consonni S Larson ED A cost-benefit analysis of black liquor gasification combined cycle systems 2003 Chicago. TAPPI Press: Engineering, Pulping & PCE&I 22 7

[B37] Jin Y Hassan J Chang H-M Green Liquor pretreatment of mixed hardwood for ethanol production in a repurposed kraft pulp mill J Wood Chem Technol 2010 30 1 86 104 10.1080/02773810903578360

[B38] Fornell R Berntsson T Process integration study of a kraft pulp mill converted to an ethanol production plant. Part A: Potential for heat integration of thermal separation units Appl Thermal Eng 2012 35 81 90

